# Functional tracts of the cerebellum—essentials for the neurosurgeon

**DOI:** 10.1007/s10143-020-01242-1

**Published:** 2020-02-13

**Authors:** Thomas Beez, Christopher Munoz-Bendix, Hans-Jakob Steiger, Daniel Hänggi

**Affiliations:** Department of Neurosurgery, Medical Faculty, Heinrich-Heine-University, Moorenstrasse 5, 40225 Düsseldorf, Germany

**Keywords:** Neuroanatomy, Cerebellar mutism syndrome, Posterior fossa, Children, Medulloblastoma

## Abstract

The cerebellum is historically implicated in motor coordination, but accumulating modern evidence indicates involvement in non-motor domains, including cognition, emotion, and language. This correlates with the symptoms observed in postoperative cerebellar mutism syndrome (CMS). Profound knowledge of cerebellar functional topography and tractography is important when approaching cerebellar tumors, as surgical trauma to relevant structures of cerebellar pathways plays a role in the pathogenesis of CMS. The aim of this systematic review is to provide a concise overview of relevant modern neuroimaging data and cerebellar functional tracts with regard to neurosurgical procedures.

## Introduction

**Fig. 1:**
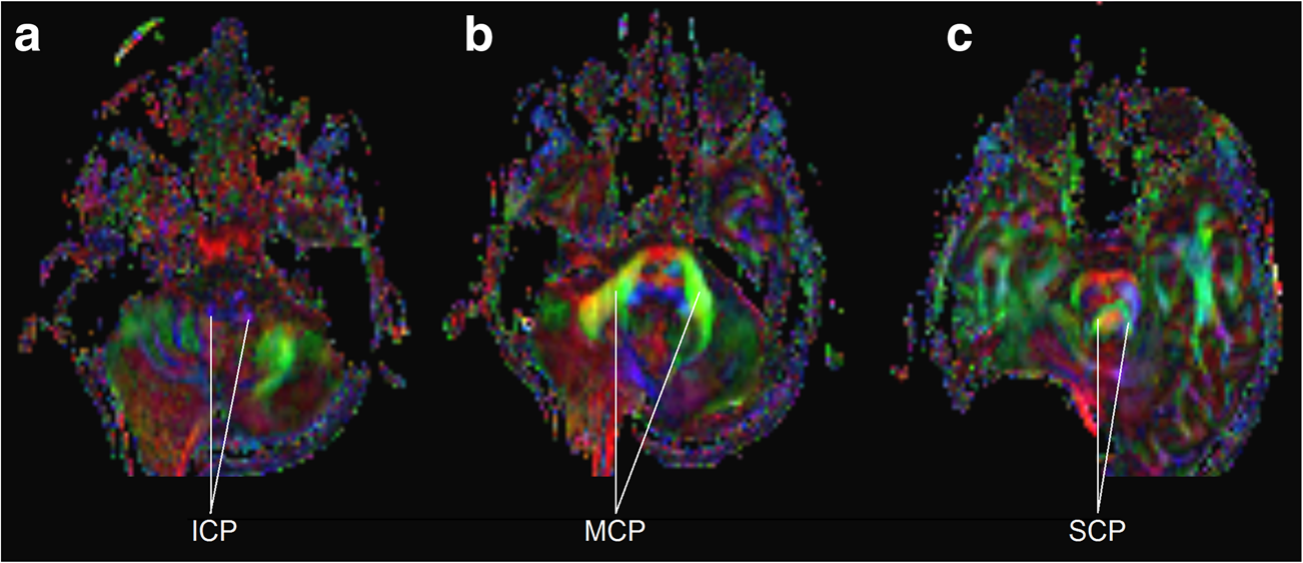
Diffusion tensor imaging of a 12-year-old boy with pilocytic astrocytoma with axial sections demonstrating the inferior (**a**), middle (**b**), and superior (**c**) cerebellar peduncles. The right sided image distortion results from a shunt valve artifact

The cerebellum is a frequent localization of brain tumors, especially in the pediatric age group. Resections of such tumors are nowadays characterized by low overall neurological morbidity rates, with routinely performed intraoperative neuromonitoring of cranial nerves and cerebrospinal pathways being an important factor [38]. However, despite modern approaches to cerebellar tumors, some sequelae are not completely preventable: postoperative cerebellar mutism syndrome (CMS) occurs in 10–25% of children, with the highest incidence apparently after medulloblastoma resections [9]. While there is consensus on the definition of this syndrome, its etiology and pathophysiology remain unclear [13]. CMS is clinically characterized by delayed onset of mutism and emotional lability, which can occur in combination with muscular hypotonia, dysphagia, as well as cerebellar cognitive affective syndrome, and cerebellar motor syndrome [13]. Although modern publications refined the identification of risk factors or at least revealed correlations, the incidence of CMS appears to be stable over time according to preliminary results from an ongoing prospective study [12, 45]. This observation could be explained by incomplete understanding of the exact pathophysiology and/or inability to effectively monitor and protect the relevant anatomical structures at risk during surgery. These structures at risk are the cerebellar nuclei and cerebellar peduncles [26], due to their high probability of playing a role in CMS, their intimate proximity to most posterior fossa tumors and their (currently) low electrophysiological monitorability during surgery. Compared with supratentorial functional systems, such as the primary motor cortex and the corticospinal tract, cerebellar functional anatomy is rarely considered in detail in general neurosurgical practice [30]. However, modern neuroimaging provides structural (e.g., diffusion tensor imaging, DTI) and functional (e.g., functional magnetic resonance imaging, fMRI) data on the cerebellum (Fig. 1) [4]. Recent fMRI studies revealed distinct functional subregions activated by motor, working memory, language, social, and emotional tasks, not always congruent with the classical anatomical lobular subdivisions [14, 22]. The aim of the current review is to provide a scientifically sound and accessible overview of modern knowledge on cerebellar functional connectivity relevant for a broad neurosurgical audience.

## Materials and methods

Following the “Preferred Reporting Items for Systematic Reviews and Meta-Analyses” (PRISMA) guidelines, a systematic PubMed search was performed for the terms “cerebellum” and “tractography” in literature published between January 2000 and December 2017 [29]. Results were manually filtered for studies using structural and functional magnetic resonance imaging (MRI) methods on healthy humans and provided explicitly connectivity information on the following nodes: (1) cerebellar localization, (2) cerebellar afferent or efferent pathway, and (3) extracerebellar localization. As the publications were heterogeneous with regard to definitions and reported parameters, pooling of data at the level of individual subjects was not feasible. With regard to the present study’s pragmatic aim, we thus compiled a quantitative descriptive analysis of N studies reporting each functional connection. Cerebellar cortical nomenclature is based on the proposal by Stoodley and Schmahmann [42]. Data was processed using GraphPad Prism for Windows (GraphPad Software, San Diego, USA) and visualized in a circular layout using Circos [23].

## Results

**Fig. 2:**
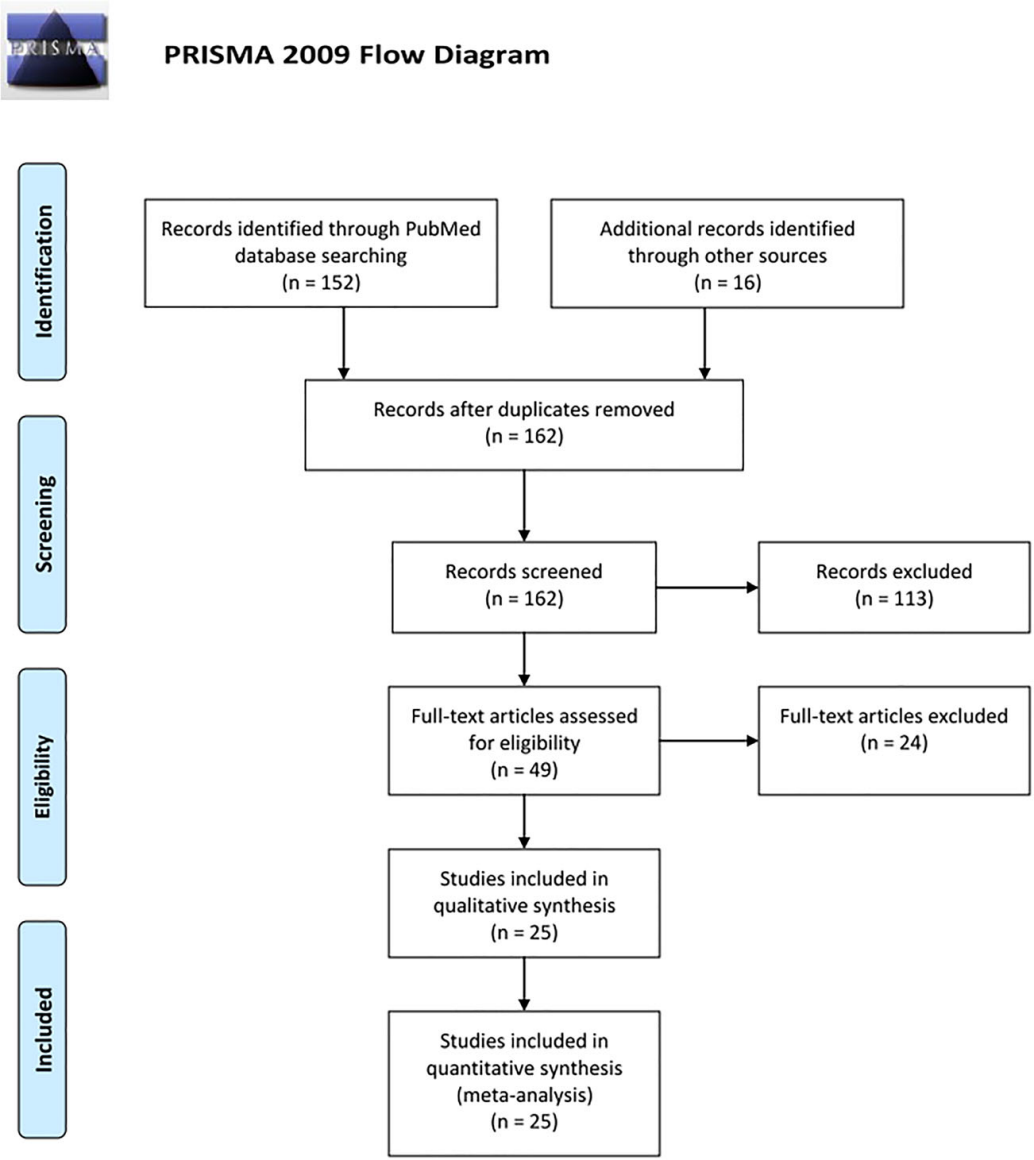
PRISMA flow diagram of the present study

From 162 records, ultimately 25 studies were included in the analysis, providing MRI-based connectivity data for 1917 subjects with an age range from 30 gestational weeks to 87 years (Fig. 2) [2, 3, 7, 8, 10, 11, 15–17, 19–21, 24, 25, 28, 31, 33–37, 39, 40, 43, 44]. Fifteen studies reported sensorimotor connectivity [2, 7, 10, 15, 19, 21, 24, 28, 33, 35–37, 39, 43, 44]. Cognitive, associative, and limbic connectivities were described in 14 studies [3, 7, 8, 17, 20, 21, 31, 34, 35, 37, 39, 40, 43, 44].

**Fig. 3:**
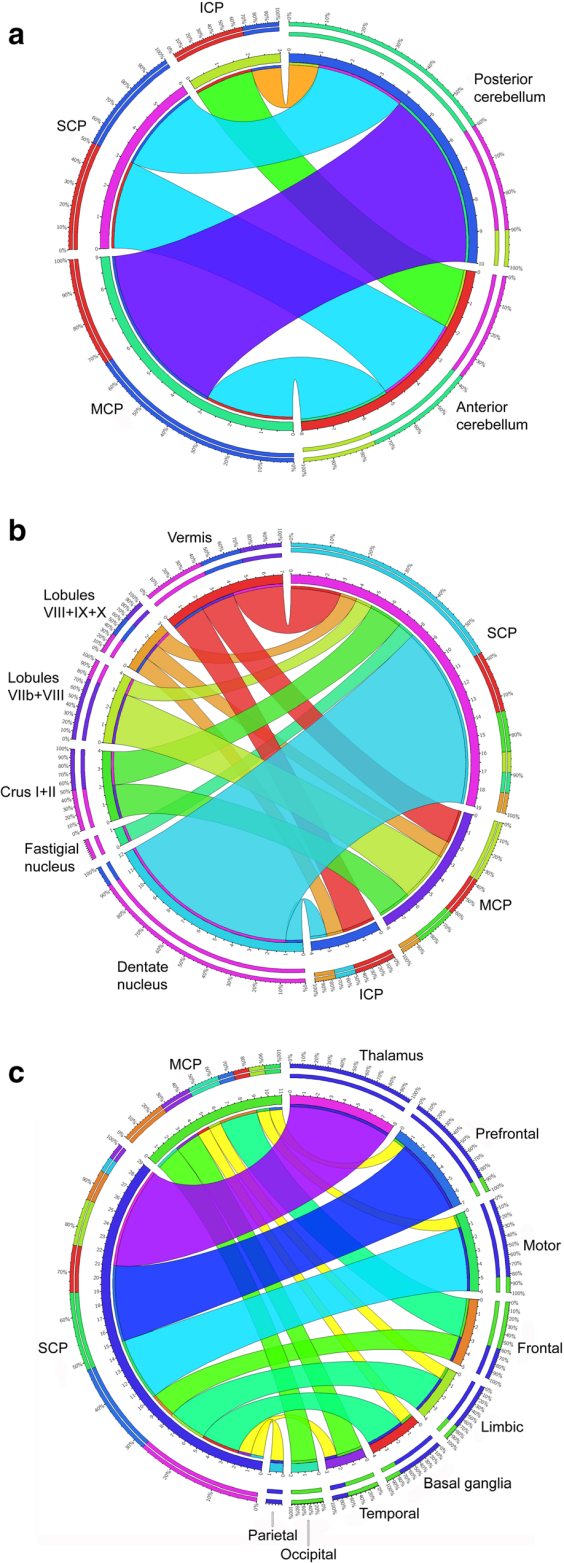
Circular chart visualization of the connectivity between dichotomized functional cerebellar region and cerebellar peduncles (**a**), cerebellar cortical and nuclear anatomical areas and cerebellar peduncles (**b**), and cerebellar peduncles and supratentorial anatomical areas (**c**)

As visualized in Fig. 3a, connectivity of the anterior cerebellum, which is predominantly involved in sensorimotor function, was reported in similar frequency with the superior cerebellar peduncle (SCP), middle cerebellar peduncle (MCP), and inferior cerebellar peduncle (ICP). For the posterior cerebellum, which is mainly associated with cognitive, emotional, and limbic functions, connectivity was reported mainly for the MCP, a lesser frequency for the SCP, and only rarely for the ICP.

The vast majority of reported connections of the dentate nucleus (DN) were projections via the SCP, and to a smaller extent via the ICP (Fig. 3b). Direct connectivity of the MCP to several cerebellar cortical locations was evident, reflecting corticocerebellar and pontinocerebellar tracts.

With regard to connectivity to supratentorial cerebral structures (Fig. 3c), the SCP was found to connect mainly to thalamus as well as prefrontal and primary motor cortex. Connectivity of the MCP was reported for a broad spectrum of cerebral areas, including cortex, basal ganglia, and limbic system (but not thalamus). At the level of the brainstem, the pontine, red, and inferior olivary nuclei are implicated in these connections.

## Discussion

This systematic review of structural and functional MRI data confirms a basic dichotomy in the human cerebellum, with sensorimotor functions being located in the anterior cerebellum and cognitive, emotional, and limbic functions found in the posterior cerebellum. For both functional areas, broad connectivity to supratentorial structures via the cerebellar peduncles was confirmed. The SCP is connected to the DN and thalamus, reflecting the dentate (rubro) thalamic tract. The MCP is connected to the posterior cerebellum and a variety of supratentorial areas reflecting the pontinocerebellar tracts. The ICP is connected to the anterior cerebellum, reflecting spinocerebellar and olivocerebellar tracts.

**Fig. 4:**
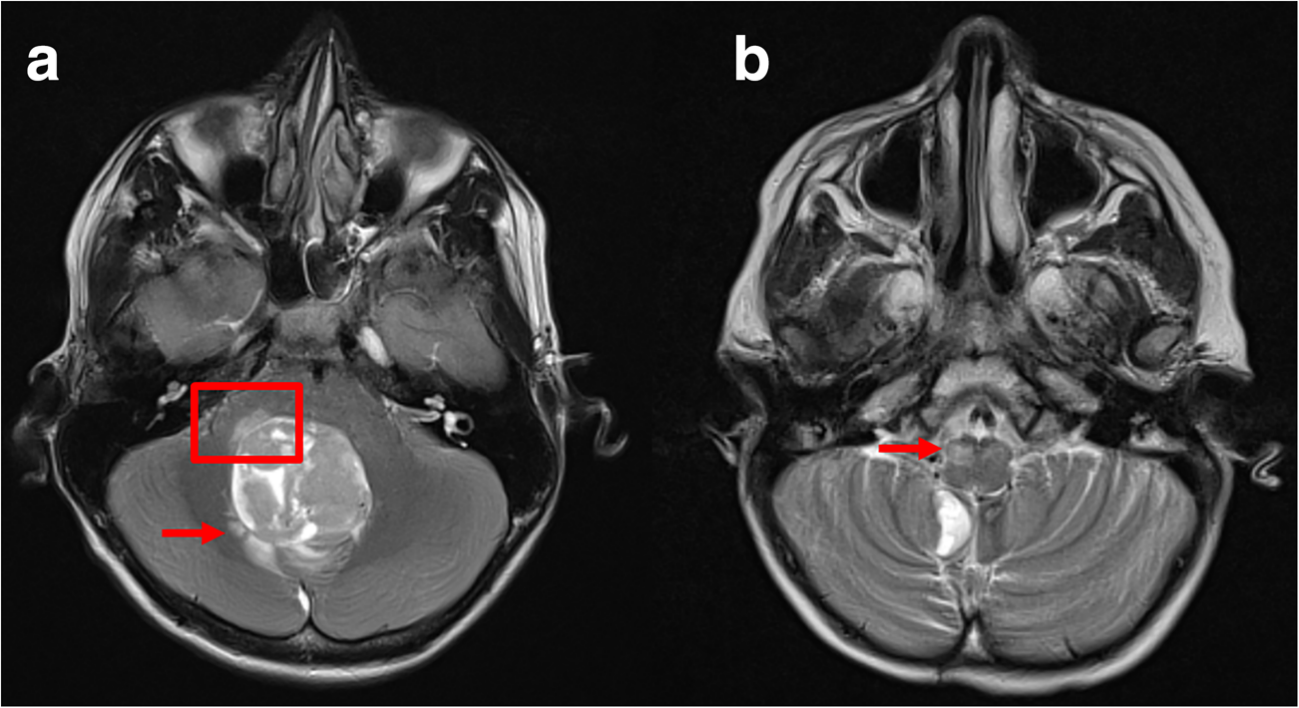
T2-weighted magnetic resonance imaging in the axial plane. Panel **a** shows preoperative imaging of a medulloblastoma with perifocal edema extending into the right middle cerebellar peduncle (red box) and right dentate nucleus (red arrow). Panel **b** demonstrates right-sided hypertrophic olivary degeneration (red arrow) occurring 5 months after medulloblastoma resection as a morphological correlate of postoperative cerebellar mutism

The widespread connectivity and topography of the human cerebellum with cerebral areas implicated in non-motor functions was demonstrated in a previous activation-likelihood meta-analysis of neuroimaging studies investigating cerebellar activation in language, verbal working memory, spatial and executive function, as well as emotional processing [42]. This involvement in several functional systems as well as the topographic dichotomy is clinically relevant, as patients suffering cerebellar stroke develop predominantly motor versus cognitive deficits depending on the location of the ischemic damage in the posterior versus anterior cerebellum [41]. For CMS, the exact localization of functional disruption remains unclear. The dentato-thalamo-cortical pathway has been implicated to play a role in CMS [6, 32]. Moreover, hypertrophic olivary degeneration (HOD) has been observed in children suffering CMS (Fig. 4b), which generally indicates injury of the dentato-rubro-olivary pathway and occurs after different types of primary insults, including ischemic stroke [5, 27]. In the context of CMS, the occurrence of HOD appears to be a surrogate parameter for damage to the DN and cerebellar pathways in general. The underlying functional anatomic correlate is the triangle of Guillain and Mollaret, which comprises the DN, ICP, and SCP [27]. Anatomical studies have emphasized the close spatial relationship between the DN and the cerebellar peduncles with its fiber tracts terminating at or surrounding the DN [1]. From an anatomical point of view, the risk of DN injury is reduced in the telovelar and sub tonsillar approaches compared with the transvermian approach [1]. From a technical point of view, thermal injury due to the use of cavitron ultrasonic surgical aspirators (CUSA) and bipolar coagulation as well as surgical retraction might be mechanisms putting the relevant anatomical structures at risk (Fig. 4a) [30].

The risk of developing CMS is higher in children compared with adults. A higher incidence of posterior fossa tumors and higher vulnerability of the developing brain are possible explanations. However, the anatomical substrate might be very similar, as certain characteristics described in the literature review by Ildan et al. are similar when comparing children and adults [18]. The higher incidence of CMS in medulloblastoma patients is a striking resemblance: 31% of patients in the adult CMS cohort had a medulloblastoma, whereas medulloblastoma accounts for only 1% of overall adult brain tumor cases. This notion is supported by Wibroe et al. who found linguistic impairments in 16% of adult patients after posterior fossa surgery [46].

Considering the findings of this systematic review, the landmark papers on functional topography of the cerebellum, the data derived from pathological entities other than CMS sharing similar symptoms, and the indirect evidence generated by clinical and imaging studies of CMS patients, we conclude that cerebellar functional anatomy should receive similar awareness during infratentorial surgery as for instance motor or language systems during supratentorial tumor resections. Based on this assumption, further insight into the pathophysiology of CMS enhancing preoperative individual risk assessment as well as intraoperative measures to protect the DN and cerebellar pathways (e.g., by optimal surgical approach, avoidance of mechanical and thermal injury, and developing specific neuromonitoring) are required. A better insight into possible presurgical deficits due to disruption of cerebellar functional systems by the tumor itself can further enhance risk stratification. For instance, invasion of cerebellar peduncles is sometimes observed on initial imaging of medulloblastoma (Fig. 4a). In the age of molecular medicine, as we are accumulating knowledge of molecular tumor subtypes and their implications for prognosis, prediction of the risk of developing CMS might be one factor guiding the aggressiveness of the surgical treatment as part of a personalized approach to each patient.

Limitations of this study are mainly related to the pragmatic study design. Due to considerable heterogeneity with regard to methodology, definitions, and reported parameters, pooling of data at the level of individual subjects was not feasible. By performing a quantitative descriptive analysis of N studies reporting each functional connection irrespective of cohort size, the results represent the number of studies reporting each connectivity as a surrogate parameter rather than the actual anatomical size of the tract. Although this might introduce bias, we nevertheless consider the results sufficient with regard to the study’s aim, i.e., to provide a stimulating overview of modern knowledge on cerebellar functional connectivity relevant for a broad neurosurgical audience.

## Conclusion

This systematic review provides a pragmatic summary of the literature on functional connectivity of the cerebellum focused on a tractographic approach most relevant from a neurosurgical perspective. Cerebellar involvement in sensorimotor function and in cognitive, associative, and limbic functions correlates with the complex and widely scattered symptoms found in CMS, including the disruption of higher cerebral functions. In addition to the cerebellar cortex, the DN, MCP, and SCP are major posterior fossa structures involved in cerebellar connectivity. During infratentorial tumor resections, these structures cannot be reliably monitored at present, but are at risk due to their proximity and exposure. Certainly, the dogma of the cerebellum being merely responsible for motor coordination is historical and has to be abandoned, especially in the operating room when approaching a cerebellar or fourth ventricular tumor.

## References

[1] Akakin A, Peris-Celda M, Kilic T, Seker A, Gutierrez-Martin A, Rhoton A (2014). The dentate nucleus and its projection system in the human cerebellum: the dentate nucleus microsurgical anatomical study. Neurosurgery.

[2] Aravamuthan BR, Muthusamy KA, Stein JF, Aziz TZ, Johansen-Berg H (2007). Topography of cortical and subcortical connections of the human pedunculopontine and subthalamic nuclei. Neuroimage.

[3] Arrigo A, Mormina E, Anastasi GP, Gaeta M, Calamuneri A, Quartarone A, De Salvo S, Bruschetta D, Rizzo G, Trimarchi F, Milardi D (2014). Constrained spherical deconvolution analysis of the limbic network in human, with emphasis on a direct cerebello-limbic pathway. Front Hum Neurosci.

[4] Avula S (2019) Radiology of post-operative paediatric cerebellar mutism syndrome. Childs Nerv Syst:1–9. 10.1007/s00381-019-04224-x 10.1007/s00381-019-04224-x 31183530

[5] Avula S, Spiteri M, Kumar R, Lewis E, Harave S, Windridge D, Ong C, Pizer B (2016). Post-operative pediatric cerebellar mutism syndrome and its association with hypertrophic olivary degeneration. Quant Imaging Med Surg.

[6] Van Baarsen KM, Grotenhuis JA (2014). The anatomical substrate of cerebellar mutism. Med Hypotheses.

[7] van Baarsen KM, Kleinnijenhuis M, Jbabdi S, Sotiropoulos SN, Grotenhuis JA, van Cappellen van Walsum AM (2016). A probabilistic atlas of the cerebellar white matter. Neuroimage.

[8] Cacciola A, Milardi D, Calamuneri A, Bonanno L, Marino S, Ciolli P, Russo M, Bruschetta D, Duca A, Trimarchi F, Quartarone A, Anastasi G (2017). Constrained spherical deconvolution tractography reveals cerebello-mammillary connections in humans. Cerebellum.

[9] Catsman-Berrevoets CE (2017). Cerebellar mutism syndrome. Curr Opin Neurol.

[10] García-Gomar MG, Soto-Abraham J, Velasco-Campos F, Concha L (2017). Anatomic characterization of prelemniscal radiations by probabilistic tractography: implications in Parkinson’s disease. Brain Struct Funct.

[11] Granziera C, Schmahmann JD, Hadjikhani N, Meyer H, Meuli R, Wedeen V, Krueger G (2009). Diffusion spectrum imaging shows the structural basis of functional cerebellar circuits in the human cerebellum in vivo. PLoS One.

[12] Grønbæk J, Keating R, Wisoff J, Juhler M (2019) Introduction to the cerebellar mutism syndrome. Childs Nerv Syst:1–2. 10.1007/s00381-019-04298-7 10.1007/s00381-019-04298-7 31399763

[13] Gudrunardottir T, Morgan AT, Lux AL, Walker DA, Walsh KS, Wells EM, Wisoff JH, Juhler M, Schmahmann JD, Keating RF, Catsman-Berrevoets C (2016). Consensus paper on post-operative pediatric cerebellar mutism syndrome: the Iceland Delphi results. Childs Nerv Syst.

[14] Guell X, Gabrieli JDE, Schmahmann JD (2018). Triple representation of language, working memory, social and emotion processing in the cerebellum: convergent evidence from task and seed-based resting-state fMRI analyses in a single large cohort. Neuroimage.

[15] Guye M, Parker GJM, Symms M, Boulby P, Wheeler-Kingshott CAM, Salek-Haddadi A, Barker GJ, Duncan JS (2003). Combined functional MRI and tractography to demonstrate the connectivity of the human primary motor cortex in vivo. Neuroimage.

[16] Habas C, Cabanis EA (2007). Anatomical parcellation of the brainstem and cerebellar white matter: a preliminary probabilistic tractography study at 3 T. Neuroradiology.

[17] Habas C, Cabanis EA (2007). Cortical projection to the human red nucleus: complementary results with probabilistic tractography at 3 T. Neuroradiology.

[18] Ildan F, Tuna M, Erman T, Göçer AI, Zeren M, Cetinalp E (2002). The evaluation and comparison of cerebellar mutism in children and adults after posterior fossa surgery: report of two adult cases and review of the literature. Acta Neurochir.

[19] Jang SH, Kwon HG (2014). Diffusion tensor tractography for the dorsal spinocerebellar tract in the human brain. Somatosens Mot Res.

[20] Jissendi P, Baudry S, Balériaux D (2008). Diffusion tensor imaging (DTI) and tractography of the cerebellar projections to prefrontal and posterior parietal cortices: a study at 3T. J Neuroradiol.

[21] Kamali A, Kramer LA, Frye RE, Butler IJ, Hasan KM (2010). Diffusion tensor tractography of the human brain cortico-ponto-cerebellar pathways: a quantitative preliminary study. J Magn Reson Imaging.

[22] King M, Hernandez-Castillo CR, Poldrack RA, Ivry RB, Diedrichsen J (2019). Functional boundaries in the human cerebellum revealed by a multi-domain task battery. Nat Neurosci.

[23] Krzywinski M, Schein J, Birol I, Connors J, Gascoyne R, Horsman D, Jones SJ, Marra MA (2009). Circos: an information aesthetic for comparative genomics. Genome Res.

[24] Kwon HG, Hong JH, Hong CP, Lee DH, Ahn SH, Jang SH (2011). Dentatorubrothalamic tract in human brain: diffusion tensor tractography study. Neuroradiology.

[25] Law N, Greenberg M, Bouffet E, Laughlin S, Taylor MD, Malkin D, Liu F, Moxon-Emre I, Scantlebury N, Skocic J, Mabbott D (2015). Visualization and segmentation of reciprocal cerebrocerebellar pathways in the healthy and injured brain. Hum Brain Mapp.

[26] Liu JF, Dineen RA, Avula S, Chambers T, Dutta M, Jaspan T, MacArthur DC, Howarth S, Soria D, Quinlan P, Harave S, Ong CC, Mallucci CL, Kumar R, Pizer B, Walker DA (2018). Development of a pre-operative scoring system for predicting risk of post-operative paediatric cerebellar mutism syndrome. Br J Neurosurg.

[27] Macht S, Hänggi D, Turowski B (2009). Hypertrophic olivary degeneration following pontine cavernoma hemorrhage: a typical change accompanying lesions in the Guillain-Mollaret triangle. Klin Neuroradiol.

[28] Meola A, Comert A, Yeh FC, Sivakanthan S, Fernandez-Miranda JC (2016). The nondecussating pathway of the dentatorubrothalamic tract in humans: human connectome-based tractographic study and microdissection validation. J Neurosurg.

[29] Moher D, Liberati A, Tetzlaff J, Altman DG (2009). Preferred reporting items for systematic reviews and meta-analyses: the PRISMA statement. PLoS Med.

[30] Molinari E, Pizer B, Catsman-Berrevoets C, Avula S, Keating R, Paquier P, Wisoff JH, Walsh KS, Posterior Fossa Society (2019) Posterior Fossa Society consensus meeting 2018: a synopsis. Childs Nerv Syst:1–7. 10.1007/s00381-019-04220-1 10.1007/s00381-019-04220-1 31177321

[31] Mollink J, van Baarsen KM, Dederen PJWC, Foxley S, Miller KL, Jbabdi S, Slump CH, Grotenhuis JA, Kleinnijenhuis M, van Cappellen van Walsum AM (2016). Dentatorubrothalamic tract localization with postmortem MR diffusion tractography compared to histological 3D reconstruction. Brain Struct Funct.

[32] Morris EB, Phillips NS, Laningham FH, Patay Z, Gajjar A, Wallace D, Boop F, Sanford R, Ness KK, Ogg RJ (2009). Proximal dentatothalamocortical tract involvement in posterior fossa syndrome. Brain.

[33] Muthusamy KA, Aravamuthan BR, Kringelbach ML, Jenkinson N, Voets NL, Johansen-Berg H, Stein JF, Aziz TZ (2007). Connectivity of the human pedunculopontine nucleus region and diffusion tensor imaging in surgical targeting. J Neurosurg.

[34] Nioche C, Cabanis EA, Habas C (2009). Functional connectivity of the human red nucleus in the brain resting state at 3T. Am J Neuroradiol.

[35] Palesi F, Tournier JD, Calamante F, Muhlert N, Castellazzi G, Chard D, D’Angelo E, Wheeler-Kingshott CAM (2015). Contralateral cerebello-thalamo-cortical pathways with prominent involvement of associative areas in humans in vivo. Brain Struct Funct.

[36] Pelzer EA, Hintzen A, Goldau M, von Cramon DY, Timmermann L, Tittgemeyer M (2013). Cerebellar networks with basal ganglia: feasibility for tracking cerebello-pallidal and subthalamo-cerebellar projections in the human brain. Eur J Neurosci.

[37] Re TJ, Levman J, Lim AR, Righini A, Grant PE, Takahashi E (2017). High-angular resolution diffusion imaging tractography of cerebellar pathways from newborns to young adults. Brain Behav.

[38] Roth J, Korn A, Sala F, Benvenisti H, Jubran M, Bitan-Talmor Y, Ekstein M, Constantini S (2019) Intraoperative neurophysiology in pediatric supratentorial surgery: experience with 57 cases. Childs Nerv Syst:1–10. 10.1007/s00381-019-04356-0 10.1007/s00381-019-04356-0 31422426

[39] Salmi J, Pallesen KJ, Neuvonen T, Brattico E, Korvenoja A, Salonen O, Carlson S (2010). Cognitive and motor loops of the human cerebro-cerebellar system. J Cogn Neurosci.

[40] Sillery E, Bittar RG, Robson MD, Behrens TEJ, Stein J, Aziz TZ, Johansen-Berg H (2005). Connectivity of the human periventricular-periaqueductal gray region. J Neurosurg.

[41] Stoodley CJ, MacMore JP, Makris N, Sherman JC, Schmahmann JD (2016). Location of lesion determines motor vs. cognitive consequences in patients with cerebellar stroke. NeuroImage Clin.

[42] Stoodley CJ, Schmahmann JD (2009). Functional topography in the human cerebellum: a meta-analysis of neuroimaging studies. Neuroimage.

[43] Takahashi E, Song JW, Folkerth RD, Grant PE, Schmahmann JD (2013). Detection of postmortem human cerebellar cortex and white matter pathways using high angular resolution diffusion tractography: a feasibility study. Neuroimage.

[44] Thomas Yeo BT, Krienen FM, Sepulcre J, Sabuncu MR, Lashkari D, Hollinshead M, Roffman JL, Smoller JW, Zöllei L, Polimeni JR, Fisch B, Liu H, Buckner RL (2011). The organization of the human cerebral cortex estimated by intrinsic functional connectivity. J Neurophysiol.

[45] Wibroe M, Cappelen J, Castor C, Clausen N, Grillner P, Gudrunardottir T, Gupta R, Gustavsson B, Heyman M, Holm S, Karppinen A, Klausen C, Lönnqvist T, Mathiasen R, Nilsson P, Nysom K, Persson K, Rask O, Schmiegelow K, Sehested A, Thomassen H, Tonning-Olsson I, Zetterqvist B, Juhler M (2017). Cerebellar mutism syndrome in children with brain tumours of the posterior fossa. BMC Cancer.

[46] Wibroe M, Rochat P, Juhler M (2018). Cerebellar mutism syndrome and other complications after surgery in the posterior fossa in adults: a prospective study. World Neurosurg.

